# Kaempferol’s Therapeutic Applications and Mechanistic Insights in Ocular Diseases: Current Progress, Challenges, and Translational Opportunities

**DOI:** 10.3390/pharmaceutics18080996

**Published:** 2026-08-12

**Authors:** Zhirui Ma, Dazheng Zhang, Xinyu Chen, Fuwen Zhang

**Affiliations:** 1 Eye School of Chengdu University of Traditional Chinese Medicine, Chengdu 610075, China; mazhirui@stu.cdutcm.edu.cn (Z.M.);; 2 Eye Health with Traditional Chinese Medicine Key Laboratory of Sichuan Province, Chengdu 610075, China

**Keywords:** kaempferol, ocular diseases, ophthalmic drug delivery systems, retinal diseases, corneal neovascularization, molecular mechanisms, safety

## Abstract

Kaempferol is a natural flavonol compound widely present in various single-herb remedies and compound formulations used for the treatment of ocular diseases. Despite its inherent pharmaceutical limitations, accumulating evidence indicates that kaempferol exerts broad protective effects against diverse ocular disorders through multiple biological pathways, highlighting its potential as a multi-target therapeutic candidate in ophthalmology. However, current evidence regarding kaempferol-based ophthalmic applications remains fragmented across different ocular diseases and mechanistic investigations, and a comprehensive evaluation of its therapeutic potential, translational challenges, and existing limitations is still lacking. This review systematically summarizes the research progress on kaempferol in the treatment of eye diseases, encompassing its source distribution, structural characteristics, ocular delivery strategies, disease spectrum coverage, molecular mechanisms, and safety profile. By critically evaluating currently available evidence, this review further identifies unresolved issues and translational barriers that hinder the clinical application of kaempferol in ophthalmology. Regarding delivery strategies, carriers such as gelatin nanoparticles, porous bovine serum albumin membranes, platelet-derived extracellular vesicles, and polyvinylpyrrolidone-based nanocomposites have preliminarily improved ocular surface retention and corneal permeability of kaempferol in models of corneal neovascularization and alkali burns. In terms of therapeutic indications, kaempferol has demonstrated protective effects in diverse experimental models, including age-related macular degeneration (AMD), diabetic retinopathy, diabetic cataract, dry eye disease, fungal keratitis, corneal transplant rejection, acute glaucoma, and retinoblastoma. At the mechanistic level, kaempferol exerts comprehensive pharmacological actions—anti-inflammatory, antioxidant, metabolic regulation, anti-angiogenic, and immunomodulatory—by modulating multiple signaling pathways, including MAPK, NF-κB, STAT1/IRF7, Nrf2/HO-1, VEGF/PI3K/Src/Akt/ERK, aldose reductase, estrogen-related receptor alpha (ERRα), and the NOD-like receptor family pyrin domain-containing protein 3 (NLRP3) inflammasome. Available safety assessments suggest that kaempferol exhibits a generally favorable safety profile across ocular, cellular, systemic, and genetic evaluations. Despite these advances, the clinical translation of kaempferol in ophthalmology remains limited by insufficient clinical and pharmacokinetic evidence, underdeveloped targeted delivery strategies, and a lack of integrated understanding of its molecular basis in ocular protection. By systematically integrating evidence from ocular disease models, molecular mechanisms, delivery strategies, and safety evaluations, this review bridges fragmented knowledge regarding kaempferol-based ophthalmic applications and provides an integrated framework for understanding its therapeutic potential and translational prospects. Overall, this review highlights kaempferol as a promising multi-target therapeutic candidate for ocular diseases and provides insights into its future translational development.

## 1. Introduction

Ocular diseases represent a major cause of visual impairment and reduced quality of life [1], and their prevalence continues to rise under the dual pressures of population aging and metabolic disorders [2,3]. This growing burden constitutes an urgent public health challenge. Currently, most ophthalmic medications have inherent limitations [4,5]; however, as the understanding of disease pathogenesis deepens, the development of safe, multi-target natural compounds has emerged as a key direction in ophthalmic drug discovery [6,7]. Kaempferol, a natural flavonoid compound, is widely present in single-herb remedies and compound Chinese herbal preparations used for the treatment of ocular diseases [8,9]. Numerous studies have demonstrated that kaempferol exhibits anti-inflammatory, antioxidant, anti-angiogenic, and immunomodulatory activities, exerting protective effects across diverse experimental models of ocular diseases [10,11,12,13,14,15]. However, a systematic synthesis and integration of existing research findings in this field remains lacking. Therefore, this review aims to systematically summarize current knowledge regarding the therapeutic potential of kaempferol in ocular diseases.

To overcome the inherent physicochemical limitations of kaempferol—including poor water solubility and low ocular bioavailability—ocular delivery strategies have shown initial promise in improving its potential for ophthalmic applications. For example, Chuang et al. [16] developed kaempferol-loaded gelatin nanoparticles (GNP-KA), which effectively inhibited corneal neovascularization in mice following topical ocular administration. Nevertheless, a systematic synthesis of relevant research is urgently needed to clarify the scope of applicability and guide future optimization efforts.

At the level of disease models and mechanisms of action, kaempferol has demonstrated therapeutic potential in multiple ocular disease models. For age-related macular degeneration, Noguchi et al. [11] confirmed in a light-induced retinal injury mouse model that kaempferol protected photoreceptor cell structure and maintained retinal function; Du et al. [10] reported in a hydrogen peroxide-induced retinal pigment epithelial (RPE) cells model that kaempferol reduced reactive oxygen species (ROS) levels, inhibited apoptosis, and suppressed inflammatory factors; for diabetic retinopathy (DR), Zhang et al. [17] observed in diabetic animal models that kaempferol alleviated retinal vascular leakage and apoptosis; for ocular surface diseases, Chen et al. [18] combined kaempferol with ferulic acid to prepare eye drops, which increased tear secretion and repaired the corneal epithelium in a rabbit model of dry eye syndrome; for *Aspergillus fumigatus* keratitis, Jia et al. [14] demonstrated in a mouse model that kaempferol reduced inflammation and fungal burden by inhibiting the Dectin-1 and p38 mitogen-activated protein kinase (MAPK) pathways. Although the aforementioned studies have revealed the multi-target pharmacological properties of kaempferol from various disease perspectives, the existing evidence remains fragmented, with insufficient cross-comparison and systematic integration across the implicated mechanisms of action and disease models.

**Figure 1:**
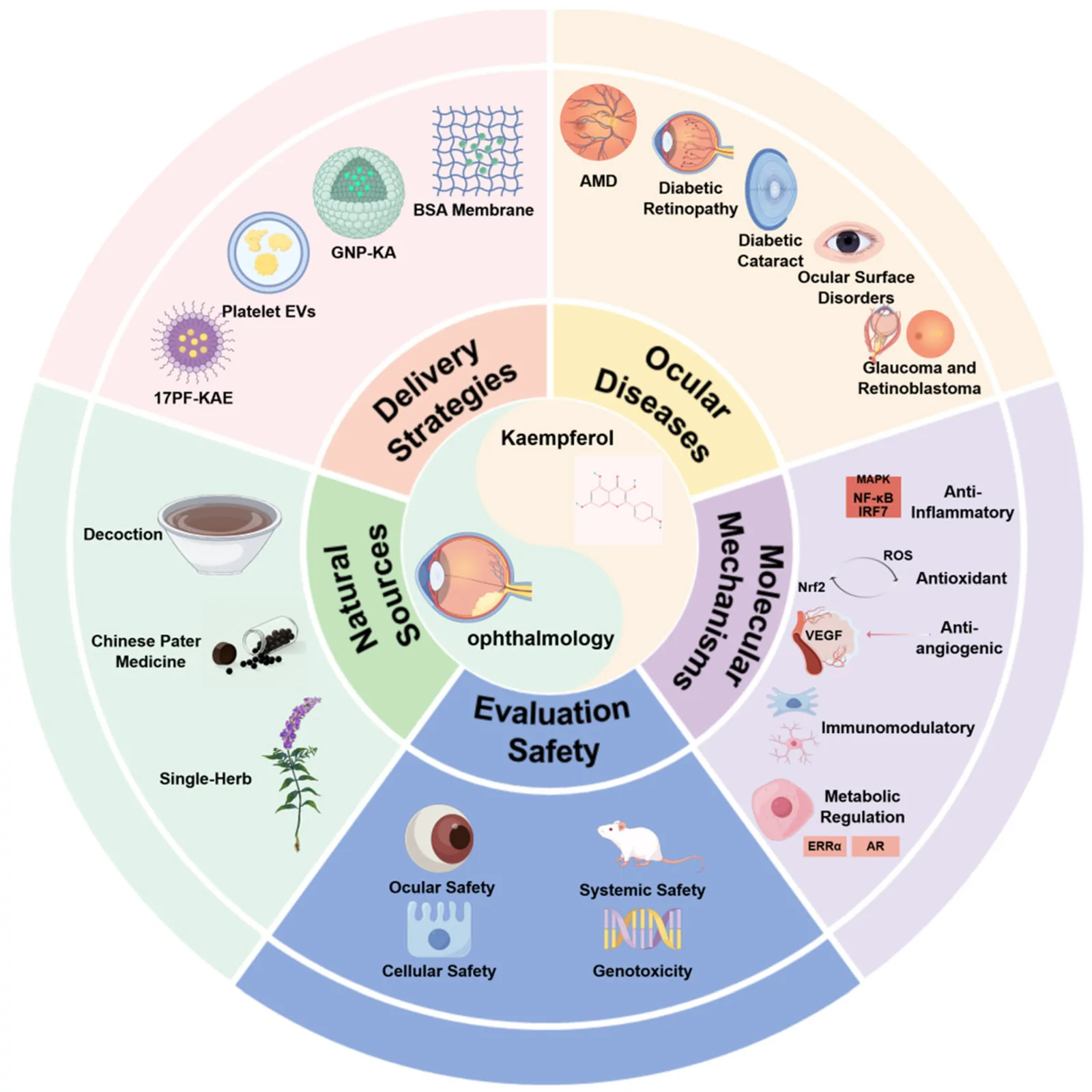
Graphical abstract: Comprehensive landscape of kaempferol in ophthalmic diseases: disease spectrum, molecular mechanisms, natural sources, delivery strategies, and safety considerations. Note: EVs, extracellular vesicles; GNP-KA, gelatin nanoparticles loaded with kaempferol; 17PF-KAE, polyvinylpyrrolidone K-17PF and kaempferol nanocomplexes; BSA, bovine serum albumin; MAPK, mitogen-activated protein kinase; NF-κB, nuclear factor kappa-B; IRF7, interferon regulatory factor 7; ROS, reactive oxygen species; Nrf2, nuclear factor erythroid 2-related factor 2.

As illustrated in Figure 1, this review is organized according to the following logic. First, it briefly introduces the source, structural characteristics, and existing ophthalmic drug delivery strategies of kaempferol, highlighting its physicochemical limitations and delivery bottlenecks. Second, it systematically summarizes the efficacy and mechanisms of action categorized by disease type, exploring their commonalities and differences. Finally, it evaluates the safety profile of kaempferol and, in light of current research gaps, proposes key scientific questions that need to be addressed in future studies, thereby providing a systematic reference for subsequent in-depth research and clinical translation.

## 2. Kaempferol Characteristics, Sources, and Ophthalmic Delivery

### 2.1. Structural Characteristics

**Figure 2:**
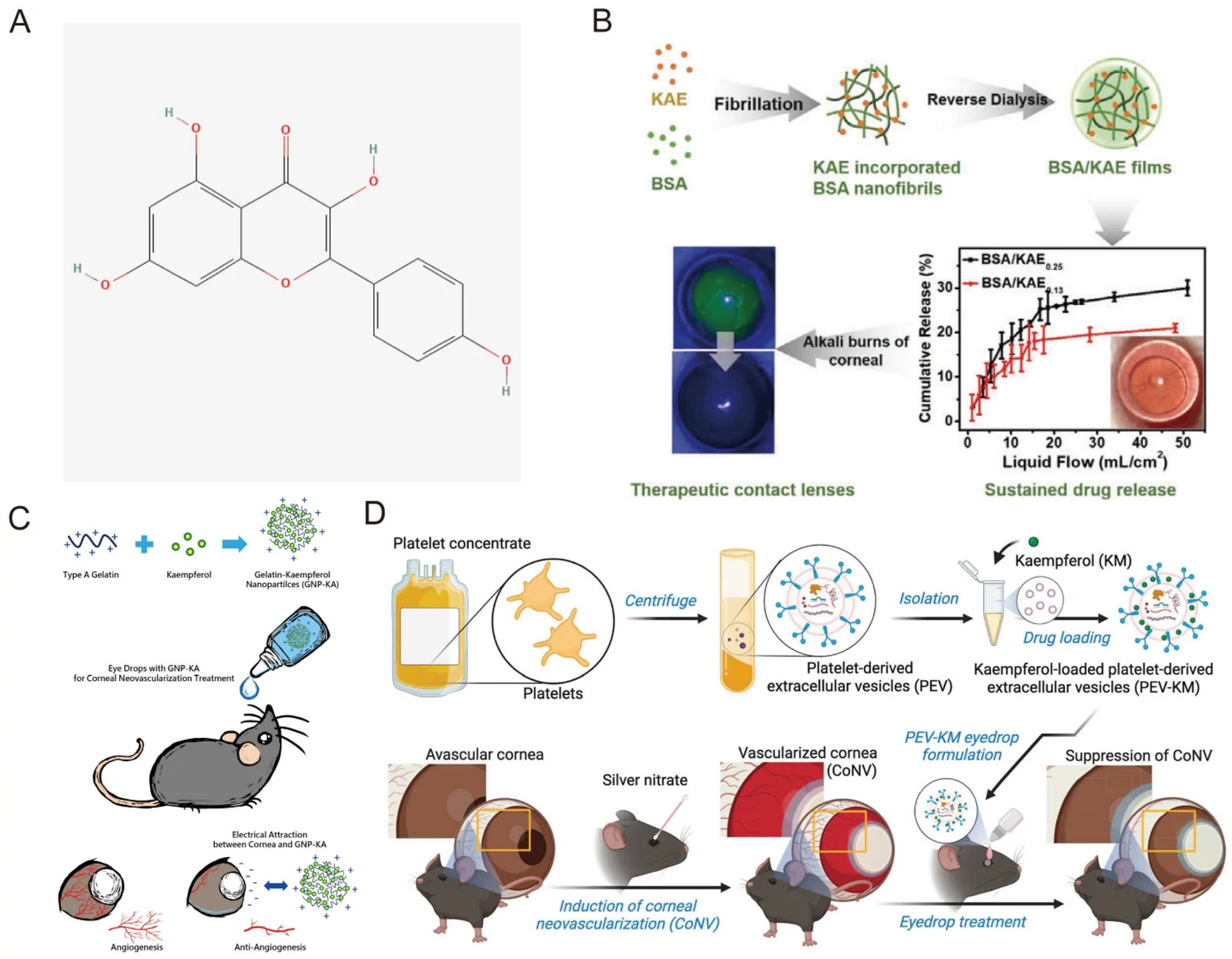
Chemical structure of kaempferol and representative ocular delivery strategies. Note: (**A**) 2D chemical structure of kaempferol (**B**) Kaempferol-incorporated BSA fibrous films for ocular drug delivery [31] © 2021 Wiley-VCH GmbH. (**C**) Kaempferol-loaded platelet-derived extracellular vesicle eye drops (PEV-KM) for ocular delivery [32] © 2025 The Authors. Published by Elsevier Ltd. (**D**) Kaempferol-loaded gelatin nanoparticles (GNP-KA) for the treatment of corneal neovascularization in mice [16] © 2020 Elsevier B.V. All rights reserved.

The chemical structure of kaempferol has been systematically characterized from multiple perspectives. According to standard chemical nomenclature, this compound belongs to the tetrahydroxyflavone class, with a molecular formula of C_15_H_10_O_6_ and a molecular weight of 286.24 g/mol [19,20], as shown in Figure 2A. The four hydroxyl groups are located at positions 3, 5, 7, and 4′ on the flavonoid skeleton, and the melting point ranges from 276 to 278 °C. Kaempferol is slightly soluble in water but exhibits good solubility in hot ethanol and ethanol [21,22]. Based on the established basic skeleton, theoretical calculations and spectroscopic analyses have further clarified its intramolecular hydrogen bonding network and spectral characteristics. Milenković et al. [23] conducted density functional theory calculations and infrared spectroscopy studies, revealing the formation of a stable six-membered ring intramolecular hydrogen bond between C_4_=O and C_5_-OH, which results in the ^1^H Nuclear Magnetic Resonance (NMR) signal of C_5_-OH appearing near δ 12.5 in deuterated dimethyl sulfoxide (DMSO-d_6_). This signal is a diagnostic feature of 5-hydroxyflavonoid compounds [24]. Moreover, the stretching vibration of C_4_=O is observed at approximately 1660 cm^−1^, while the four hydroxyl groups produce a broad and strong overlapping absorption band in the 2900–3500 cm^−1^ region. This broad band has been resolved as the superposition of four O-H stretching vibrations and an overtone mode [25]. Correlation coefficient and mean absolute error analyses of theoretical calculations versus experimental values for ^13^C and ^1^H NMR chemical shifts, based on the GIAO method, further confirmed that B3LYP-D3 is a reliable theoretical model for describing the NMR parameters of kaempferol. Experimental synthesis studies have provided corroborating evidence from the perspective of structural modification: Deng et al. [26] introduced a sulfonic acid group at the C3′ position of kaempferol and prepared a gallium complex involving coordination with C_3_-OH and C_4_=O. By combining high-resolution mass spectrometry and infrared spectroscopy, they confirmed that C_3_-OH and C_4_=O are the most reactive sites in the parent molecule, thereby indirectly confirming that the 3-OH and 4-CO groups constitute a chelating unit in the flavonol skeleton. Together, this information—encompassing intramolecular hydrogen bonding, characteristic NMR chemical shifts, diagnostic infrared absorptions, and reactive sites—constitutes a comprehensive understanding of the chemical structure of kaempferol. These structural characteristics directly determine the various biological activities of kaempferol [27,28,29,30].

Together, this information—encompassing intramolecular hydrogen bonding, characteristic NMR chemical shifts, diagnostic infrared absorptions, and reactive sites—constitutes a comprehensive understanding of the chemical structure of kaempferol. These structural features underpin its pharmacological activities, including anti-inflammatory, antioxidant, and anti-angiogenic effects, which account for its widespread presence in traditional medicines for ophthalmic use.

### 2.2. Natural Source

Kaempferol, a natural flavonoid compound, is widely distributed in fruits and vegetables [33,34] and constitutes one of the principal active ingredients in many ophthalmic drugs [8,35]. Among single-herb Chinese medicine formulations, the following have been reported: *Astragalus membranaceus* for the treatment of DR [36]; *Amelanchier lamarckii*, which exhibits protective effects in retinal pigment epithelial cell models [37]; *Ehretia asperula*, which exerts antioxidant protective effects on retinal progenitor cells [38]; *Thuja orientalis*, which reduces oxidative damage to retinal ganglion cells [39]; and *Sedum dendroideum*, which inhibits the proliferation of pterygium fibroblasts [40]. In decoction formulations—including Chufeng Yisun Decoction [41], YiqiYangyin Decoction [42], Si-Ni-San [43], and Mimeng Flower Decoction [44]—kaempferol has been identified as a common active ingredient. Similarly, in traditional Chinese patent medicines, including Zhangyanming Tablet [45], Compound-Xueshuantong Capsule [46], and ShuangdiShouzhen Tablet [47], kaempferol is also recognized as one of the important active components.

In summary, kaempferol is abundantly present in a wide range of traditional Chinese medicine formulations and ophthalmic drugs, underscoring its broad availability as a natural source for ocular therapeutic development.

### 2.3. Delivery Strategies in Ophthalmology

Despite the pharmacological activity conferred by its natural scaffold, kaempferol’s structural characteristics also give rise to significant physicochemical limitations [48]. Although kaempferol exhibits good biocompatibility and membrane permeability [49,50] and holds significant potential for detection, diagnostic, and therapeutic applications [51,52], it is hampered by poor water solubility, low corneal permeability, and chemical instability in alkaline aqueous media [53,54,55]. At the pharmacokinetic level, Peng et al. [56] employed a validated UPLC-MS/MS method to quantify kaempferol distribution in rat ocular tissues following oral administration, revealing that only 0.0041% of the administered dose reached ocular tissues at 35 min post-administration, directly demonstrating the extremely low ocular bioavailability of oral kaempferol. When administered via the topical route, the drug must traverse the corneal barrier for anterior segment targets, and the blood–retinal barrier for posterior segment targets; the cumulative effect of these multiple barriers renders unmodified kaempferol unable to achieve effective therapeutic concentrations at target sites. Taken together, both its favorable and unfavorable properties, along with its pharmacokinetic barriers, inform the optimization of its dosage forms. Current research on the ocular application of kaempferol-based biomaterials primarily focuses on the use of natural and synthetic carrier materials to deliver kaempferol for the treatment of ocular diseases.

In terms of naturally derived biomaterials for drug loading, current carriers primarily include animal-derived biomaterials such as gelatin, bovine serum albumin (BSA), and EVs. Chuang et al. [16] prepared gelatin nanoparticles encapsulating kaempferol (GNP-KA) with a particle size of 85–150 nm and an encapsulation efficiency of 90–98%. Topical administration via eye drops effectively inhibited corneal neovascularization in mice and reduced the production of matrix metalloproteinases and vascular endothelial growth factor (VEGF) in the cornea, as shown in Figure 2D. However, in vitro release kinetics, corneal permeability (Papp), and long-term storage stability were not characterized, and the use of GA cross-linking warrants further biocompatibility evaluation. Furthermore, Yin et al. [31] incorporated kaempferol into porous BSA films to form transparent, high-water-content BSA/KAE fibrous films. These films achieved sustained drug release over 340 h in artificial tears, promoted corneal re-epithelialization, inhibited neovascularization, and reduced inflammation in a corneal alkali burn model, demonstrating superior efficacy compared to kaempferol alone, as shown in Figure 2B. However, Papp was not quantified and storage stability of the drug-loaded state was not reported. At the nanocarrier level, Liu et al. [32] constructed platelet-derived EVs loaded with kaempferol (PEV-KM), which had an average diameter of approximately 160 nm and an encapsulation efficiency of about 61%. Topical administration via eye drops prolonged the ocular retention time and achieved sustained release. However, Papp and long-term storage stability remain to be evaluated. Compared with PEV alone, the combination with kaempferol exhibited stronger effects in inhibiting angiogenesis and downregulating pro-angiogenic and pro-inflammatory cytokines in an alkali burn-induced corneal neovascularization model, as shown in Figure 2C. Regarding synthetic materials, Zhang et al. [57] designed ultra-small nanocomposites (17PF-KAE) based on polyvinylpyrrolidone, with a size of approximately 8.6 nm. These nanocomposites exhibited good aqueous dispersibility and corneal permeability, significantly increasing the concentration of kaempferol in the cornea and aqueous humor in a mouse model of corneal alkali burns. Notably, 17PF-KAE was the only system for which corneal Papp was quantified and storage stability was rigorously evaluated. The therapeutic mechanism was associated with the inhibition of intercellular adhesion molecule-1 (CD54), interleukin-6 (IL-6), transforming growth factor-beta 1 (TGF-β1) and VEGF. Additionally, Chaudhuri et al. [58] loaded kaempferol into a pH-triggered in situ gel-forming system. This system gelled in response to the ocular pH environment following eye drop administration, significantly prolonging the drug retention time on the ocular surface. It demonstrated a sustained pharmacological response and good ocular tolerance in a sodium selenite-induced rat cataract model, providing a novel formulation strategy for the non-invasive treatment of cataracts with kaempferol. A comprehensive comparison of pharmaceutics parameters—including loading mechanism, encapsulation efficiency, release kinetics, corneal permeability, ocular retention, and storage stability—across these delivery systems is provided in Table 1.

Overall, kaempferol delivery systems—built from animal-derived biomaterials (gelatin, BSA, platelet EVs) and synthetics (polyvinylpyrrolidone, pH-triggered gels)—serve as useful tools for corneal neovascularization, alkali burns, and cataracts. Table 1 compares four systems (pH gel excluded due to missing quantitative data). Physically entrapped GNP-KA gives highest encapsulation but lacks release/permeability data; BSA film enables ultra-long release but misses quantitative characterization; PEV-KM excels in ocular retention with lower encapsulation; 17PF-KAE alone provides Papp and stability despite simpler encapsulation. So far, only two systems have release kinetics, one has corneal Papp, and one has stability data—pointing to an early-stage field. Most carriers also lack scalability, long-term safety, and biocompatibility data. Therapeutically, these systems improve stability, retention, permeability, and sustained release.

Yet most studies focus on corneal neovascularization and alkali burns, with little exploration of cataracts, glaucoma, DR, or AMD. Also, delivery research stays largely in the anterior segment, leaving the posterior understudied. DR and AMD pose extra barriers—blood-retinal barriers, oxidative stress, and inflammation—so strategies must be tailored. For DR, nanocarriers targeting retinal endothelial cells (with anti-VEGFR2 or RGD) can boost transport across the inner BRB, while ROS-sensitive systems allow on-demand release in hyperglycemic conditions. For AMD, ligands like elastin-like polypeptides or integrin-binding motifs can target RPE and Bruch’s membrane, combined with sustained-release platforms (microspheres or hydrogels) to improve subretinal accumulation. Beyond eye drops, routes like subtenon, intravitreal, and suprachoroidal injections need systematic pharmacokinetic and safety evaluation. Clearly, we need multidimensional strategies that integrate active targeting, microenvironment responsiveness, and optimized delivery to extend kaempferol from anterior to posterior diseases.

Future research should address: (i) systematic pharmaceutics characterization, particularly release kinetics, Papp, and storage stability, across all delivery systems; (ii) clinical translation potential including scale-up feasibility and sterilization; (iii) multifunctional nanoplatforms combining active targeting and controlled release; and (iv) clinically relevant intraocular administration routes with pharmacokinetic evaluation. (v) Targeted delivery strategies for posterior segment diseases, including DR and AMD, should be developed and evaluated, with emphasis on active targeting ligand modification, microenvironment-responsive drug release, and appropriate administration routes, to fill the current research gaps. Such efforts will enable systematic assessment of the efficacy and safety of kaempferol-based formulations, thereby laying a foundation for their practical application in the clinical treatment of ocular diseases.

## 3. Kaempferol in Ocular Disease Therapy

Ocular diseases encompass organic and functional lesions of various tissue structures of the eye, including the retina, cornea, lens, uvea, optic nerve, and intraocular tumors. Research on kaempferol in ocular diseases has covered age-related macular degeneration, diabetic eye diseases, ocular surface diseases, as well as glaucoma and retinoblastoma, as shown in Table 2. This section will elaborate on each of these diseases individually.

**Table 2:** Kaempferol in ocular disease therapy.

Disease Category	Specific Disease/Model	Experimental Model	Dosage and Frequency	Administration Route	Key Findings	Ref.
Age-related macular degeneration	Light-induced retinal injury	BALB/c mice (light injury)	20 mg/kg, once daily for 5 days after light injury	Intraperitoneal injection	Counteracts light-induced retinal dysfunction, structural thinning, and apoptosis.	[11]
	H_2_O_2_-induced RPE cell injury	ARPE-19 cells (H_2_O_2_-induced oxidative stress)	50 μM, 24 h pretreatment, then 300 μM H_2_O_2_ for 24 h	In vitro	Increases cell survival rate and effectively alleviates cellular damage.	[59]
Diabetic eye diseases	diabetic retinopathy	C57BL/6 mice (STZ-induced)	10 mg/kg (LD)/30 mg/kg (HD), once daily for 8 weeks	Oral	Protects retinal structure and vessels, and ameliorates retinal cell apoptosis.	[17]
		HRECs (high-glucose-induced)	10/30 μM in 30 mM glucose for 24 h	In vitro	Protects HRECs against high-glucose-induced proliferation, migration, and tube formation.	[60]
	Diabetic cataract	Goat lens organ culture (high-glucose-induced)	50 μM with 30 mM glucose	In vitro	Inhibits lens protein glycation and aldose reductase activity.	[61]
		SRA01/04 (high-glucose-induced EMT)	5/20 μg/mL in 30 mM glucose	In vitro	Inhibits high-glucose-induced epithelial–mesenchymal transition in lens epithelial cells.	[62]
Ocular surface diseases	dry eye syndrome	C57/BL6 mice (BAC-induced)	25 mg/kg (LD)/75 mg/kg (HD), once daily for 4 weeks	Oral	Improves tear film stability and protects against corneal surface damage.	[63]
	Corneal neovascularization	C57BL/6 mice (alkali burn-induced)	GNP-KA eye drops (KA 7.05 μg/mL), once daily for 7 days	Eye drops	Protects mouse cornea, reduces neovascularization area, and prolongs ocular surface retention.	[16]
	Corneal transplant rejection	SD rat donors, Wistar rat recipients	50 mg/kg, once daily, from day −3 to sacrifice	Intraperitoneal injection	Effectively alleviates postoperative rejection.	[15]
	Bacterial keratitis	C57BL/6 mice (*S. aureus*-induced)	5% (*w*/*v*) compound 52, 4 times daily for 3 days	Topical eye drops (compound 52)	Protects mouse cornea and reduces bacterial burden.	[64]
	*Aspergillus fumigatus* keratitis	C57BL/6 mice (*A. fumigatus*-induced)	i.p: 1 mL of 60 μg/mL KAE; subconj.: 5 μL; once daily, day 1–3 p.i.	Intraperitoneal + subconjunctival injection	Protects mouse cornea and reduces fungal load.	[14]
Other diseases	Acute glaucoma	C57BL/6 mice (I/R)	100 mg/kg, single dose (sacrificed at 48 h)	Oral	Preserves retinal thickness and reduces retinal ganglion cell death.	[13]
	Retinoblastoma	Human retinoblastoma SO-RB50 cells	30 μM for 24 h	In vitro	Inhibits proliferation and invasion of retinoblastoma cells.	[65]

Note: RPE, retinal pigment epithelial; HRECs, human retinal endothelial cells; SRA01/04, a human lens epithelial cell line; EMT, epithelial–mesenchymal transition; BAC, Benzalkonium Chloride; I/R, ischemia-reperfusion; i.p., intraperitoneal injection; LD, low dose; HD, high dose; STZ, streptozotocin; BAC, benzalkonium chloride; I/R, ischemia/reperfusion; GNP-KA, gelatin nanoparticle-encapsulated kaempferol; p.i., post-infection.

### 3.1. Age-Related Macular Degeneration

Age-related macular degeneration (AMD) is a leading cause of blindness in the elderly population worldwide [66]. The disease is broadly classified into two forms. The dry (atrophic) form is characterized by progressive degeneration of retinal pigment epithelial (RPE) cells and secondary loss of photoreceptors [67]; the wet (neovascular) form is driven by choroidal neovascularization (CNV) [68]. Kaempferol, a multifunctional natural flavonoid, has been shown to regulate key pathological features of retinal diseases [69,70,71,72,73]. Its anti-inflammatory, antioxidant, and anti-apoptotic activities align closely with two critical pathogenic mechanisms in dry AMD—RPE degeneration and photoreceptor death. These properties position kaempferol as a candidate of growing interest for AMD intervention. This section evaluates the existing evidence along these two dimensions and identifies the translational gaps that must be addressed before clinical application can be contemplated.

**Figure 3:**
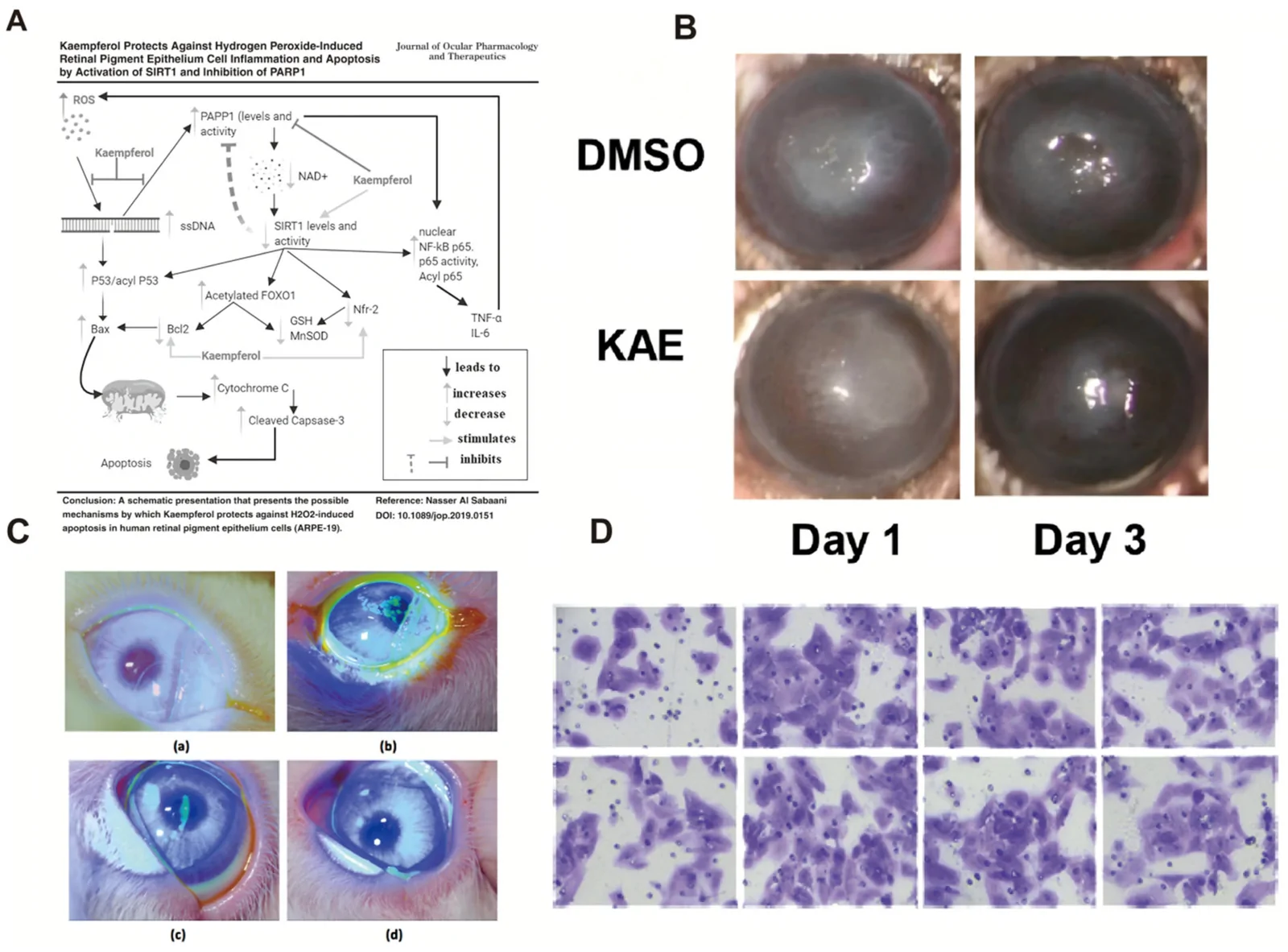
Protective effects of kaempferol against multiple ocular tissue injuries. Note: (**A**) Kaempferol protects retinal pigment epithelial cells against H_2_O_2_-triggered inflammation and apoptosis through the SIRT1/PARP1 pathway [59]. © Mary Ann Liebert, Inc.; (**B**) Representative corneal images of mice infected with *Aspergillus fumigatus* at 1 and 3 days post-infection after treatment with DMSO or kaempferol. The images show the progression of corneal lesions and the protective effects of kaempferol against fungal keratitis–associated opacity, ulceration, and inflammatory changes [14]. © 2022 Elsevier Ltd. All rights reserved. (**C**) FA100/KM1 significantly improves and repairs BAC-induced rabbit corneal epithelial injury [18] © 2017 by the authors. Licensee MDPI, Basel, Switzerland. (**D**) Kaempferol inhibits high glucose-induced migration and invasion of SRA01/04 cells [62]. Rights managed by Taylor & Francis.

RPE cell protection. RPE dysfunction and death are early and rate-limiting events in dry AMD pathogenesis. Du et al. [10] demonstrated that in H_2_O_2_-induced oxidative damage models of RPE cells, kaempferol significantly increased cell viability and reduced the apoptotic rate. Al Sabaani et al. [59] independently confirmed that kaempferol attenuates the H_2_O_2_-induced decline in RPE cell viability and mitigates DNA damage, identifying SIRT1 activation and PARP1 inhibition as the underlying molecular axis, as shown in Figure 3A. In a lipopolysaccharide (LPS)-induced inflammatory injury model, Zhang et al. [74] further observed that kaempferol suppressed RPE cell apoptosis. Since oxidative stress and chronic low-grade inflammation are the principal drivers of RPE degeneration in dry AMD, these convergent findings from independent groups and distinct injury models provide a coherent cellular-level rationale for kaempferol’s potential application. Beyond the effects of kaempferol as a single agent, Wu et al. [70] isolated hyperoside, quercetin, and kaempferol from Kochiae Fructus, a traditional Chinese medicine used for ophthalmic conditions. They found that all three compounds activated the Nrf2/HO-1 pathway and attenuated oxidant-induced RPE cell death. Notably, hyperoside and kaempferol synergistically enhanced the cytoprotective effect of quercetin, and the triple combination exhibited significantly superior protection compared with quercetin alone, suggesting that combining kaempferol with related flavonols may further potentiate its RPE protective efficacy.

Photoreceptor cell protection. Photoreceptor degeneration represents the final common pathway to vision loss in dry AMD. Noguchi et al. [11] showed in a light-induced photodamage mouse model—which recapitulates key features of dry AMD—that kaempferol significantly reduced photoreceptor apoptosis and preserved electroretinogram amplitudes. Of particular relevance to AMD, Laabich et al. [75] reported that kaempferol conferred substantial protection against photoreceptor death induced by A2E (N-retinylidene-N-retinylethanolamine), a bis-retinoid that accumulates in RPE lipofuscin and is a well-established pathogenic factor in dry AMD. This finding distinguishes kaempferol from flavonols that protect against generic oxidative stress but lack efficacy against A2E-mediated toxicity, thereby strengthening its AMD-specific interventional rationale.

In summary, the available evidence supports the protective potential of kaempferol against two core pathogenic processes in dry AMD: RPE oxidative damage and A2E-driven photoreceptor degeneration. However, direct evidence for efficacy against CNV in wet AMD is absent, representing a significant gap. More fundamentally, no study to date has evaluated kaempferol in AMD-specific animal models—such as aged or genetically modified AMD models—that capture the chronic, progressive nature of the human disease. The existing data derive almost exclusively from acute in vitro experiments or short-term rodent models. Key limitations include model mismatch (none of the current models recapitulate drusen biogenesis or geographic atrophy), short intervention windows (all experiments are acute and cannot simulate the decades-long course of AMD), and species differences (rodents lack a macula, which limits the translational relevance of structural endpoints). Future work should prioritize long-term intervention studies in aging or transgenic AMD models and, in parallel, establish laser-induced CNV models to systematically evaluate whether kaempferol can directly suppress choroidal neovascularization and thereby address the current void in wet AMD research.

### 3.2. Diabetic Eye Diseases

Diabetic eye diseases can affect multiple structures within the eye, with DR and diabetic cataract being the most common. Their common pathophysiological origin is chronic hyperglycemia [7]. Therefore, an ideal prevention and treatment strategy should possess the dual ability to regulate blood glucose and to counteract specific ocular damage. Studies have shown that kaempferol not only inhibits α-glucosidase to regulate blood glucose [76,77,78,79,80] but also exerts an independent protective effect on the diabetic retina and lens. The following discussion addresses two conditions: DR and diabetic cataract.

#### 3.2.1. Diabetic Retinopathy

Diabetic retinopathy is one of the most common and specific microvascular complications of diabetes [81]. It is characterized by retinal vascular damage, tissue ischemia, and pathological neovascularization, which can lead to irreversible visual impairment. Currently, anti-VEGF therapy is used as a first-line treatment; however, approximately 30–40% of patients still develop drug resistance or secondary resistance [82,83]. Kaempferol, a natural flavonoid compound, has demonstrated multi-target intervention effects on DR-related pathological processes in multiple studies and is considered a potential candidate drug for the treatment of DR [84]. Current efficacy studies are mainly based on animal experiments and in vitro cell assays.

At the animal experiment level, Zhang et al. [17] found that kaempferol ameliorated weight loss, hyperglycemia, and abnormal glucose tolerance in diabetic mice, while alleviating retinal vascular leakage, cell apoptosis, damage to various layers of the retina, and fibrotic changes. The effect of a high dose of kaempferol (30 mg/kg) was more pronounced. Compared with the positive control drugs insulin and calcium dobesilate, kaempferol exhibited favorable effects in improving metabolic indicators and protecting retinal structure.

At the in vitro cell experiment level, Wu et al. [60] simulated DR conditions using human retinal endothelial cells (HRECs) cultured under high glucose. Using CCK-8, scratch, and tube formation assays, they found that the high-glucose environment did not significantly promote HREC proliferation but markedly enhanced cell migration and tube formation ability. Kaempferol at concentrations of 10 μM and 30 μM inhibited high-glucose-induced cell migration and tube formation, thereby helping to block the abnormal migration of vascular endothelial cells and neovascularization in the early stage of DR. Xu et al. [85] also used a high-glucose-induced HREC model and confirmed via CCK-8, scratch, tube formation, and Transwell assays that kaempferol dose-dependently inhibited HREC proliferation, migration, and tube structure formation within the concentration range of 5–25 μM. This effect restricts the generation of pathological neovascularization in DR from multiple aspects.

In summary, kaempferol has demonstrated protective effects against DR-related vascular leakage, apoptosis, structural damage, and pathological angiogenesis in both animal models and cellular assays. However, the existing evidence is largely confined to vascular endothelial cells, with insufficient evaluation of other cell types that may contribute to DR pathogenesis. More critically, all in vivo data have been derived from rodent models, with no efficacy validation in large-animal models such as swine or non-human primates. Pharmacokinetic data following clinically relevant routes of administration remain scarce and lack systematic characterization. Furthermore, the retinal exposure levels of kaempferol and its metabolites, their capacity to traverse the blood–retinal barrier, and their interactions with key retinal targets have not been rigorously quantified or experimentally validated. Although cell-based, rodent, and computational studies have provided preliminary insights into its protective mechanisms, it remains unclear whether kaempferol can achieve sufficient exposure in the native retinal microenvironment to elicit target-mediated pharmacological effects. This uncertainty constitutes a critical barrier to the translational development of kaempferol for diabetic retinopathy. Without more comprehensive pharmacokinetic and pharmacodynamic data in clinically relevant models, the therapeutic feasibility of kaempferol for DR cannot be reliably assessed. Future investigations should expand the range of cell types examined, establish chronic diabetic large-animal models, and conduct systematic pharmacokinetic evaluations via clinically translatable routes of administration to validate its therapeutic potential.

#### 3.2.2. Diabetic Cataract

Diabetic cataract is characterized by lens opacity, abnormal protein aggregation, and epithelial cell dysfunction [86,87]. Kaempferol has shown potential for anti-cataract intervention [58,88,89]. To systematically evaluate its effects, researchers have obtained the following evidence using three complementary models: isolated organ, enzymatic, and cellular levels.

At the isolated lens organ culture level, Patil et al. [61] used a sugar-induced lens organ culture model and found that kaempferol significantly inhibited the development of lens opacity, while also reducing abnormal aggregation of lens proteins and the formation of advanced glycation end products (AGEs). At the enzymatic model level, Hwang et al. [90] used the rat lens aldose reductase (RLAR) assay system and found that kaempferol had a significant inhibitory effect on this enzyme. In addition, the study confirmed that kaempferol possessed 2,2-diphenyl-1-picrylhydrazyl (DPPH) radical scavenging activity and effectively reduced sorbitol accumulation in rat lenses, as shown in Figure 3D. At the lens epithelial cell level, Huang et al. [62] used high-glucose-stimulated human lens epithelial cells (SRA01/04) as a model and verified through migration and invasion assays that kaempferol significantly inhibited the high-glucose-induced enhancement of cell migration and invasion abilities, suggesting that kaempferol helps delay the abnormal activation of lens epithelial cells.

These findings suggest that kaempferol may exert multifaceted protective effects against diabetic cataract through diverse biological mechanisms. However, current evidence is predominantly derived from in vitro models and ex vivo lens organ culture systems, while in vivo validation and clinical evidence remain lacking. From a pharmacokinetic perspective, the lens is an avascular tissue, and its nutrient exchange and drug exposure rely primarily on the aqueous humor circulation. To date, quantitative data regarding the ocular exposure of kaempferol following topical or systemic administration, particularly its concentration in the aqueous humor, remain unavailable. Furthermore, the exposure–response relationship linking kaempferol levels to its anti-cataract efficacy has not yet been established. In the absence of sufficient evidence demonstrating ocular exposure and pharmacodynamic relevance, the clinical translation potential of kaempferol for diabetic cataract remains to be fully validated. Future studies should establish appropriate diabetic animal models to evaluate its therapeutic efficacy, systematically characterize its pharmacokinetic profiles in ocular fluids and tissues, and integrate multi-omics approaches to elucidate the underlying protective networks.

### 3.3. Ocular Surface Diseases

Ocular surface diseases are a group of functional disorders involving the cornea, conjunctiva, adnexa, and tear film, which severely affect visual health and quality of life [91,92]. Kaempferol, with its synergistic properties of multiple pharmacological activities and multidimensional tissue repair capabilities, exhibits potential advantages in intervening in the complex pathological network of ocular surface diseases. This section will be divided into two subsections: dry eye syndrome and corneal diseases.

#### 3.3.1. Dry Eye Syndrome

Dry eye syndrome is primarily characterized by insufficient tear secretion and tear film instability, often accompanied by ocular symptoms such as dryness, burning sensation, and foreign body sensation [93]. Kaempferol has demonstrated advantages in improving tear secretion, reducing corneal damage, and enhancing tear film stability in multiple models. Existing studies have systematically evaluated the effects of kaempferol alone or in combination with other agents on ocular surface structure and function, ranging from whole-animal to in vitro cellular levels.

At the animal model level, Chen et al. [18] utilized a rabbit model of dry eye syndrome and formulated a buffer eye drop solution containing a combination of kaempferol and ferulic acid for topical administration. The results showed a significant increase in tear secretion in the treated group, along with reduced corneal epithelial damage, as shown in Figure 3C. Corneal thickness returned to near-normal levels, and no irritative reactions were observed in the eyes. In vitro experiments confirmed that this combined regimen exerts synergistic anti-inflammatory effects in lipopolysaccharide-stimulated human corneal epithelial cells, indicating that the combination of kaempferol and ferulic acid may offer enhanced therapeutic efficacy in dry eye syndrome. Zhang et al. [63] evaluated more comprehensive ocular surface parameters in a benzalkonium chloride-induced dry eye mouse model, including tear secretion tests, tear film breakup time, conjunctival goblet cell density, and corneal terminal deoxynucleotidyl transferase dUTP nick end labeling (TUNEL) staining. They found that kaempferol treatment effectively increased tear secretion, prolonged tear film breakup time, improved conjunctival goblet cell density, reduced corneal tissue apoptosis, and repaired corneal epithelial damage. Furthermore, at the in vitro cellular level, Li et al. [94] exposed human corneal epithelial cells to a hyperosmotic environment to simulate the pathological state of dry eye syndrome. After kaempferol treatment, Ki67 staining demonstrated enhanced cell proliferation, while TUNEL staining showed a reduced rate of cell apoptosis.

In summary, kaempferol improves tear secretion, protects the corneal epithelium, and maintains ocular surface structural integrity in various dry eye syndrome model. However, several deficiencies remain in this field: first, only three small-sample studies are available, lacking large-scale animal experiments and clinical trials; second, due to model heterogeneity (rabbit, mouse, cell) and differences in drug administration routes, direct comparison of efficacy is difficult; third, none of the studies evaluated long-term safety or rebound effects after drug withdrawal. Future research should conduct rigorous randomized controlled animal experiments and advance to clinical studies, while exploring the differential efficacy of kaempferol in different dry eye subtypes to clarify the applicable population and optimal regimen.

#### 3.3.2. Corneal Diseases

Corneal diseases are diverse in type, and their onset and progression are closely associated with disruption of the normal physiological homeostasis of the cornea. Maintaining a transparent and avascular state of the cornea is fundamental to its normal function [95]; however, pathological conditions such as neovascularization, transplant rejection, and infectious keratitis can disrupt this homeostasis, leading to visual impairment [96,97]. Based on etiology, corneal diseases can be divided into two categories: non-infectious and infectious. Kaempferol has shown therapeutic potential in both types.

In non-infectious corneal diseases, corneal neovascularization and corneal transplant rejection are important causes of visual impairment, and several studies have reported the effects of kaempferol. Regarding corneal neovascularization, Chuang et al. [16] administered GNP-KA as eye drops to treat corneal neovascularization in mice. The results showed that this treatment reduced abnormal corneal blood vessel growth. Liu et al. [32] applied PEV-KM topically to a mouse model of alkali burn-induced corneal neovascularization, observing a significant reduction in vascular formation in the damaged cornea. In addition, Yin et al. [31] found that in an alkali burn-induced corneal injury model, BSA porous membranes containing kaempferol not only inhibited neovascularization but also promoted corneal re-epithelialization. Regarding corneal transplant rejection, rejection is the major threat affecting surgical success rates in high-risk patients. Tian et al. [15] administered kaempferol via intraperitoneal injection in a rat corneal allograft model and found that it effectively alleviated postoperative rejection.

Bacterial and fungal infections are important causes of corneal blindness in infectious corneal diseases. Lin et al. [64] designed and synthesized kaempferol derivatives, one of which exhibited potent antibacterial activity against Gram-positive bacteria such as methicillin-resistant *Staphylococcus aureus*, and demonstrated good in vivo efficacy in a mouse corneal infection model. Regarding fungal keratitis, Jia et al. [14] treated mouse corneas infected with *Aspergillus fumigatus* with kaempferol and found that it reduced the severity of keratitis, decreased the fungal load on the cornea, and inhibited inflammatory cell recruitment, as shown in Figure 3B.

Collectively, kaempferol has demonstrated therapeutic potential in corneal neovascularization, corneal graft rejection, and infectious keratitis. Among these indications, corneal diseases represent relatively accessible targets for kaempferol-based intervention; however, the current evidence remains largely restricted to rodent studies and has yet to establish clinical relevance. A major consideration for further development is that the beneficial effects observed in corneal neovascularization models are frequently achieved through nanocarrier-based formulations, reflecting the inherent pharmaceutical limitations of kaempferol, such as poor aqueous solubility and limited ocular surface retention. Meanwhile, the consequences of long-term or repeated administration on corneal epithelial homeostasis, ocular surface microbiota, and wound healing processes remain insufficiently characterized. Beyond monotherapy, whether kaempferol can provide additional benefits when combined with established treatments, including topical corticosteroids, calcineurin inhibitors, or anti-VEGF agents, remains an open question. Such combinations may theoretically exploit complementary mechanisms and reduce dependence on prolonged high-dose treatment, but this possibility requires further experimental validation.

### 3.4. Other Diseases

In addition to the diseases described above, research on kaempferol in ophthalmology also includes glaucoma and retinoblastoma. Currently, kaempferol has shown intervention potential in anti-tumor [98,99,100] and neuroprotective [101,102] aspects. This section will elaborate on these two diseases separately.

The progressive death of retinal ganglion cells is a key pathological link in glaucoma-induced blindness [103], and glaucoma is one of the leading causes of irreversible visual impairment worldwide [104]. The protective effect of kaempferol on retinal ganglion cells has been preliminarily explored [105,106]. In a study on acute glaucoma, Ling et al. [13] established a mouse retinal ischemia-reperfusion model and observed that after kaempferol intervention, retinal thickness was preserved and retinal ganglion cell death was significantly reduced. Regarding ocular tumors, retinoblastoma is a malignant retinal tumor in children caused by inactivation of the retinoblastoma protein (pRb) gene. Qin et al. [65] found that kaempferol concentration-dependently inhibited the growth, colony formation, and invasion ability of SO-RB50 retinoblastoma cells, and its target molecules were highly expressed in tumor tissues.

Kaempferol has shown preliminary protective effects in glaucoma-related models (retinal ganglion cell preservation) and inhibitory effects on tumor-associated behaviors in retinoblastoma models (suppression of proliferation, colony formation, and invasion). However, the translational considerations for these two indications differ substantially. Glaucoma requires long-term or lifelong management, raising concerns regarding sustained ocular exposure and cumulative toxicity, whereas retinoblastoma primarily affects infants and young children, for whom long-term safety and local tolerability are particularly critical. Therefore, the future development of kaempferol for these indications should not be based solely on further confirmation of pharmacological activity, but also on establishing whether clinically relevant exposure can be achieved while maintaining an acceptable safety profile.

## 4. Molecular Mechanisms of Kaempferol in Ocular Diseases

**Figure 4:**
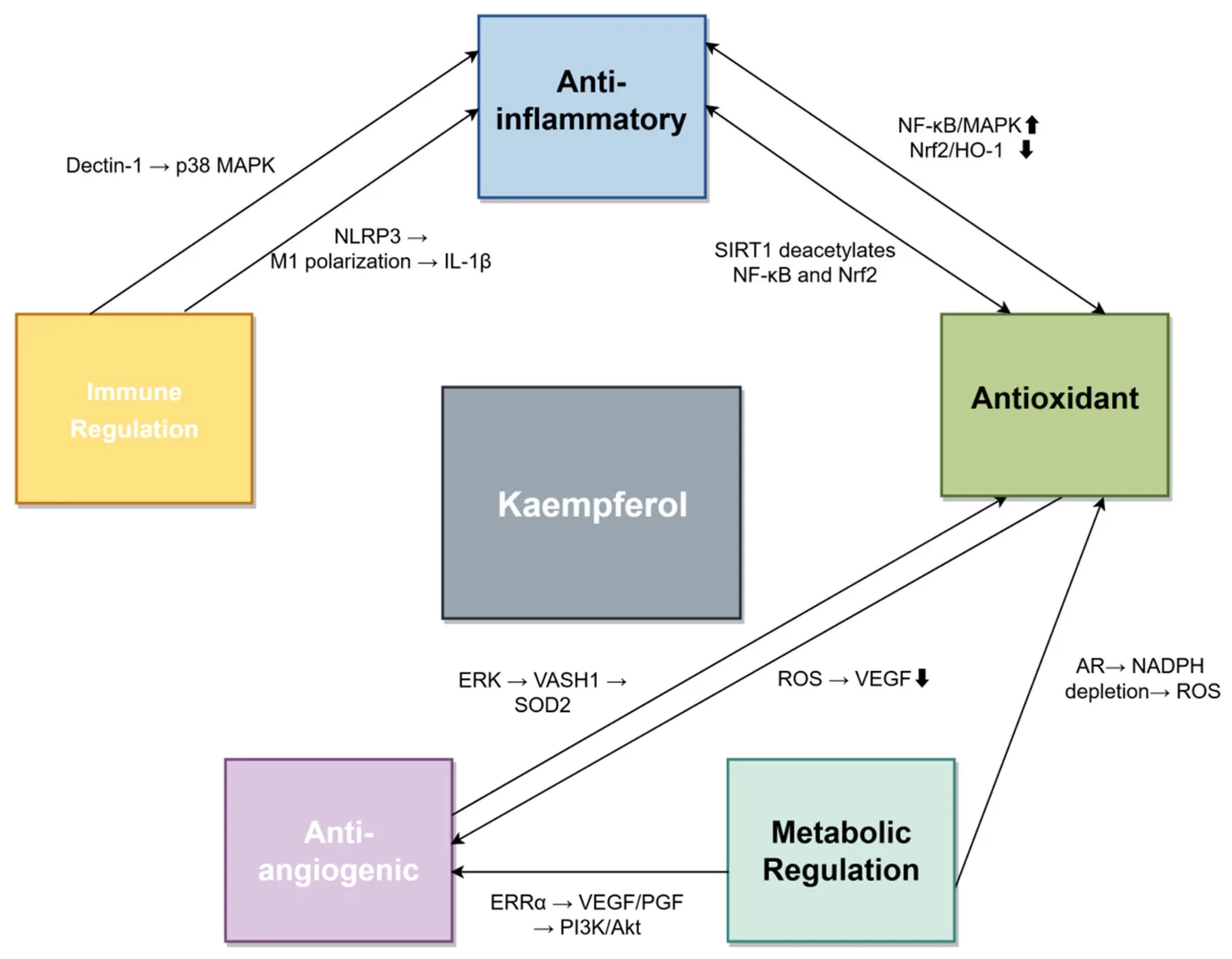
Crosstalk and hierarchical relationships among molecular mechanisms of kaempferol in ocular diseases. Notes: Arrows indicate regulatory relationships; NF-κB, nuclear factor kappa-light-chain-enhancer of activated B cells; MAPK, mitogen-activated protein kinase; ERK, extracellular signal-regulated kinase; Nrf2, nuclear factor erythroid 2-related factor 2; NLRP3, NLR family pyrin domain containing 3; SIRT1, sirtuin 1; ERRα, estrogen-related receptor alpha; Dectin-1, dendritic cell-associated C-type lectin-1; IL-1β, interleukin-1 beta; VEGF, vascular endothelial growth factor; PGF, placental growth factor; PI3K, phosphoinositide 3-kinase; Akt, protein kinase B; VASH1, vasohibin-1; SOD2, superoxide dismutase 2; AR, aldose reductase; NADPH, reduced nicotinamide adenine dinucleotide phosphate; ROS, reactive oxygen species; HO-1, heme oxygenase-1.

The protective effects of kaempferol against ocular diseases involve multiple molecular mechanisms. Existing research primarily focuses on five aspects: anti-inflammatory, antioxidant, metabolic regulation, anti-angiogenic, and immune regulation, each involving various key pathways and cellular targets, as shown in Table 3. While these five aspects are discussed individually below, they do not operate as isolated pathways. Extensive experimental evidence reveals substantial cross-talk among these mechanism modules, forming an integrated signaling network that collectively mediates the ocular protective effects of kaempferol. The key inter-module connections and their convergence hubs are summarized in Figure 4. These aspects are elaborated below.

**Table 3:** Mechanistic studies of kaempferol in ocular diseases.

Mechanism Category	Signaling Pathway/Target	Model	Validation Method	Molecular Mechanism	Ref.
Anti-inflammatory	p38 MAPK	Hyperosmolarity-induced HCECs dry eye model	qPCR, Western blot	Inhibits TNF-α, IL-6, p38 expression	[94]
	NLRP1/NLRP3 inflammasome; NF-κB pathway; JNK pathway	Retinal I/R mouse model	qPCR, Western blot	Inhibits NLRP1/NLRP3 activation; reduces IL-1β, TNF-α, IL-6, iNOS, COX-2; inhibits NF-κB p65 nuclear translocation; inhibits JNK phosphorylation	[13]
	STAT1/IRF7	LPS-induced RPE cell injury model	qPCR, Western blot, ELISA, Co-IP, GST pull-down, CHX Chase Analysis	Promotes STAT1 ubiquitination-mediated degradation of IRF7; suppresses IL-1β, IL-6 production	[74]
Antioxidant	ROS; SOD	H_2_O_2_-induced ARPE-19 oxidative stress injury	FRAP, SOD activity kit, DCFH-DA	Reduces ROS activity; increases SOD activity	[10]
	Nrf2/HO-1	Blue ligh-induced photo-oxidative stress in ARPE-19 cells	Western blot, SOD activity kit, TBARS, CMF-DA, DCFH-DA	Activates Nrf2 nuclear translocation; upregulates HO-1	[71]
Metabolic regulation	AR	RLAR in vitro assay; ex vivo rat lens high-glucose culture	RLAR enzyme activity assay, HPLC	Inhibits AR activity; reduces sorbitol accumulation	[90]
	ERRα; Wnt/β-catenin	Retinoblastoma SO-RB50 cells	Western blot, qPCR, luciferase reporter assay	Inhibits ERRα; inhibits Wnt/β-catenin	[65]
Anti-angiogenesis	VEGF/PGF	High-glucose-induced HRECs	qPCR, Western blot, ELISA, Matrigel tube formation assay	Inhibits VEGF/PGF expression; inhibits PI3K/Src/Akt1/Erk1/2	[85]
Immune regulation	NLRP3 inflammasome	LPS-stimulated HAPI microglia; BB rat type 1 DR model	qPCR, Western blot, immunofluorescence	Inhibits NLRP3 inflammasome; induces M2 polarization	[107]

Note: iNOS, inducible nitric oxide synthase. HCECs, human corneal epithelial cells; HAPI, highly aggressively proliferating immortalized; AR, aldose reductase; SO-RB50, a human retinoblastoma cell line; LPS, lipopolysaccharide.

### 4.1. Anti-Inflammatory Effects via MAPK, NF-κB and STAT1/IRF7 Pathways

Inflammation is a key common pathological link in various eye diseases [43]. Numerous studies have confirmed that kaempferol possesses significant and broad-spectrum anti-inflammatory properties [108,109], and its anti-inflammatory activity is considered one of the core pharmacological bases for its ocular protective effects [110,111,112]. In the treatment of eye diseases, its role is primarily achieved through intervention in three key inflammatory signaling pathways: MAPK, nuclear factor kappa-B (NF-κB), and signal transducer and activator of transcription 1 (STAT1)/interferon regulatory factor 7 (IRF7).

**Figure 5:**
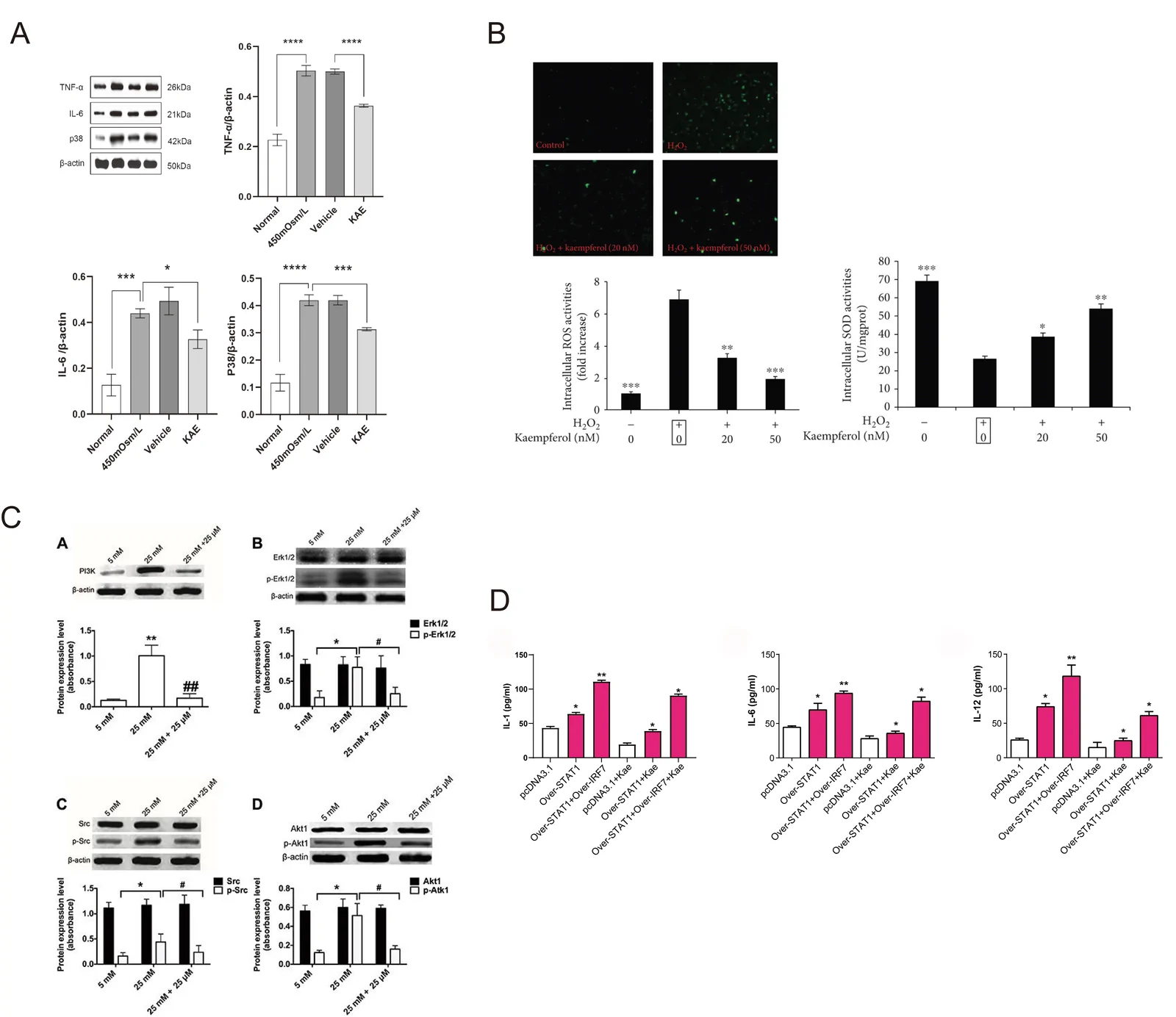
Multifaceted regulatory mechanisms of kaempferol in inflammation, oxidative stress, and angiogenesis. Note: (**A**) Kaempferol alleviates hyperosmolarity-induced ocular surface inflammation by inhibiting the p38 pathway (* *p* < 0.05, *** *p* < 0.001, and **** *p* < 0.0001) [94]. © The Author(s) 2022. (**B**) Kaempferol achieves potent antioxidant protection by reducing ROS generation and increasing SOD activity. Data were shown as mean ± SD (*n* = 3). * *p* < 0.05, ** *p* < 0.01, *** *p* < 0.001 vs. H_2_O_2_ only treated group (marked by black frame) [10]. Copyright © 2018 Weiwei Du et al. (**C**) Kaempferol blocks high-glucose-induced pro-angiogenic signaling at the molecular pathway level. Data are reported as means ± SD (*n* = 3). * *p* < 0.05, ** *p* < 0.01 compared to 5 mM. ^#^ *p* < 0.05, ^##^ *p* < 0.01 compared to 25 mM (one-way ANOVA, followed by Dunnett’s multiple comparison test) [85]. © The Author(s) 2017. (**D**) Kaempferol attenuates LPS-induced inflammatory cytokine production in RPE cells via modulation of the STAT1/IRF7 axis. * *p* < 0.05, ** *p* < 0.01 [74]. by The Authors is licensed under CC BY 4.0.

Regarding the MAPK pathway, Jia et al. [14] used a mouse model of *Aspergillus fumigatus* keratitis and found that kaempferol effectively reduces the expression of key pro-inflammatory factors such as Interleukin-1 beta (IL-1β) and tumor necrosis factor-alpha (TNF-α) in the cornea by inhibiting the phosphorylation of p38 MAPK, thereby alleviating the inflammatory damage caused by infection. Notably, this study also highlights a cross-talk between immune recognition and inflammatory signaling: fungal Dectin-1 receptor engagement serves as the upstream trigger for p38 MAPK activation, and kaempferol intervenes at this immune–inflammatory interface by inhibiting both the Dectin-1 sensor and its downstream p38 MAPK effector.Li et al. [94] confirmed in a hyperosmotic-induced dry eye syndrome model that kaempferol also exerts clear anti-inflammatory and cytoprotective effects by inhibiting the activation of the p38 MAPK pathway and downregulating the expression of TNF-α and IL-6 in human corneal epithelial cells, as shown in Figure 5A. Regarding the NF-κB pathway and related inflammasomes, Ling et al. [13] confirmed in an acute glaucoma model that kaempferol inhibits the activation of the NF-κB and c-Jun N-terminal kinase (JNK) signaling pathways, thereby blocking the assembly of downstream NLRP1 (NLR family pyrin domain containing 1)/NLRP3 (NLR family pyrin domain containing 3) inflammasomes, ultimately significantly reducing inflammation and neuronal cell death in the retina. In addition, the STAT1/IRF7 signaling axis is also a target of kaempferol. Zhang et al. [74] found that under inflammatory stimulation, kaempferol downregulates IRF7 levels by promoting the ubiquitination-mediated degradation of STAT1, thereby inhibiting the production of inflammatory factors such as IL-1β and IL-6 in retinal pigment epithelial cells, thus exerting anti-inflammatory effects at the transcriptional regulatory level.

In summary, kaempferol forms a multidimensional anti-inflammatory network by targeting multiple pathways (MAPK, NF-κB, STAT1/IRF7) in eye diseases. However, the upstream molecular targets that integrate these pathways remain unclear. However, the existing evidence is derived predominantly from acute inflammatory models in rodents; validation in chronic ocular inflammatory conditions remains absent. Future research should focus on cross-talk between pathways and the pivotal role of kaempferol within this network, which would provide a theoretical basis for developing more effective and selective kaempferol-based ocular anti-inflammatory drugs.

### 4.2. Antioxidant Defense via ROS Scavenging and Nrf2 Activation

In the pathological process of eye diseases, oxidative stress is a core cause of damage to various cells, including RPE cells and retinal ganglion cells [113,114,115], which may ultimately lead to irreversible vision loss. Currently, the antioxidant effect of kaempferol has been extensively studied and confirmed [116,117], and this effect lies at the core of its therapeutic potential. This section focuses on two main mechanisms: directly reducing ROS levels and activating the nuclear factor erythroid 2-related factor 2 (Nrf2) antioxidant signaling pathway.

In terms of directly reducing ROS levels, Du et al. [10] found that in hydrogen peroxide-induced RPE cell damage, kaempferol effectively reduces intracellular ROS activity, restores superoxide dismutase (SOD) activity, and enhances cellular antioxidant defense capacity. By simultaneously reducing ROS and increasing SOD, kaempferol helps restore the intracellular oxidative/antioxidant imbalance caused by oxidative stress, as shown in Figure 5B. Beyond its antioxidant effects, kaempferol concurrently downregulated VEGF mRNA and protein expression in both H_2_O_2_-treated ARPE-19 cells and a sodium iodate-induced retinal degeneration rat model, thereby linking the attenuation of oxidative stress to the suppression of angiogenic signaling. In addition, Zhao et al. [118] found that kaempferol dose-dependently reduces ROS accumulation in retinal ganglion cells stimulated by high glucose. Of note, the underlying mechanism involves ERK-dependent upregulation of VASH1, which promotes mitochondrial SOD2 expression—a convergence of anti-angiogenic signaling on antioxidant defense. However, the antioxidant effect of kaempferol extends far beyond direct ROS scavenging; its key feature lies in its ability to systematically activate the intracellular defense hub—the Nrf2 pathway. The mechanistic study by Al Sabaani et al. [59] demonstrates that kaempferol significantly promotes the nuclear translocation and activation of Nrf2 by activating sirtuin 1 (SIRT1) and inhibiting poly(ADP-ribose) polymerase 1 (PARP1), thereby synergistically upregulating the overall antioxidant capacity of cells. Crucially, SIRT1 activated by kaempferol concurrently deacetylates NF-κB p65 to suppress inflammatory signaling, thereby positioning this single deacetylase as a shared upstream node that bridges antioxidant defense (Nrf2) with anti-inflammatory responses (NF-κB). Liu et al. [71] provided further evidence for this, showing that at a concentration of 10 μM, kaempferol significantly increases the expression of Nrf2 in the nucleus, thereby upregulating the expression of the downstream effector molecule heme oxygenase-1 (HO-1) protein and effectively protecting RPE cells from blue light-induced photooxidative damage. Concurrently, kaempferol suppressed NF-κB protein expression and MAPK signaling in the same model, demonstrating that a single flavonoid can simultaneously engage antioxidant (Nrf2/HO-1) and anti-inflammatory (NF-κB/MAPK) transcriptional programs to counter photo-oxidative injury.

In summary, in the treatment of eye diseases, kaempferol exerts multi-level antioxidant effects by directly reducing ROS levels and activating the Nrf2 pathway. Currently, relevant research is still mainly based on in vitro experiments. Future studies should employ animal models to further clarify its antioxidant effects and dose–response relationship in vivo.

### 4.3. Metabolic Regulation via Aldose Reductase Inhibition and ERRα Antagonism

Metabolic disorders are an important pathological basis for the occurrence and development of various eye diseases [119]. Existing research indicates that kaempferol, as a natural flavonoid compound, not only inhibits key enzymes in glucose metabolism [120,121,122] but also antagonizes estrogen-related receptor alpha (ERRα) [123]. The regulatory effects of kaempferol on abnormal metabolism in eye diseases are mainly reflected in two aspects: direct inhibition of key enzymes in the glucose metabolism pathway, and regulation of the estrogen metabolism-related core transcription factor ERRα and its downstream signaling pathway.

At the level of glucose metabolism pathways, the core function of kaempferol is to inhibit aldose reductase (AR) activity and block the polyol pathway. Lee et al. [124] found that kaempferol significantly inhibited AR activity in rat lenses (IC_50_ = 1.1 μM) while reducing the formation of advanced glycation end products (AGEs) (IC_50_ = 32.1 μM). Ahmad et al. [125] revealed the direct binding mode of kaempferol to the human AR active pocket through molecular docking and dynamic simulation (binding energy of -8.16 kcal/mol) and confirmed that it acts as a mixed inhibitor. In vitro experiments further showed that kaempferol significantly reduced sorbitol accumulation in human red blood cells under high-glucose conditions (from 35.67 to 15.47 nmol/mL at 5 μM). Hwang et al. [90] also confirmed the effective inhibition of kaempferol on rat lens AR (IC_50_ = 1.11 μM) and observed its inhibitory effect on sorbitol accumulation in the lenses, demonstrating that kaempferol intercepts the polyol pathway at its enzymatic entry point—an intervention with direct implications for cellular redox balance given the NADPH-consuming nature of this pathway.

At the level of estrogen-related signaling pathway regulation, kaempferol acts as an inverse agonist of ERRα, thereby regulating downstream transcriptional networks. Wu et al. [60] found that kaempferol targeted inhibition of ERRα activity in human retinal endothelial cells cultured under high glucose, thereby downregulating vascular endothelial growth factor (VEGF) expression, upregulating the endogenous angiogenesis inhibitors thrombospondin-1 (TSP-1) and a disintegrin and metalloproteinase with thrombospondin motifs 1 (ADAMTS-1), and inhibiting cell proliferation, migration, and tube formation in a dose-dependent manner. This ERRα-VEGF regulatory axis exemplifies a direct metabolic-to-angiogenic cross-talk, in which kaempferol-mediated suppression of a metabolic transcription factor drives downstream anti-angiogenic outcomes. Qin et al. [65] confirmed in retinoblastoma cells that kaempferol inhibits the Wnt/β-catenin signaling pathway by targeting ERRα, causing cell cycle arrest and apoptosis, thereby inhibiting cell proliferation and invasion.

In summary, the metabolic regulation of kaempferol in the treatment of eye diseases is a multi-level synergistic process: it not only alleviates the direct metabolic damage mediated by the polyol pathway through upstream inhibition of AR activity but also acts as an ERRα inverse agonist to regulate downstream Wnt/β-catenin signaling pathways, affecting pathological processes such as angiogenesis, cell cycle, and apoptosis. However, the existing evidence is derived mainly from in vitro studies, with a lack of in vivo animal data, and the interaction between the AR and ERRα pathways remains unclear. Future studies should employ animal models and genetic intervention approaches to validate these findings.

### 4.4. Anti-Angiogenic Actions via VEGF-Related Signaling Pathways

Pathological ocular neovascularization is a common pathological basis of blinding eye diseases such as DR and age-related macular degeneration [126]. In recent years, multiple in vitro and in vivo experiments, as well as studies on nanoformulations, have confirmed that kaempferol exerts a clear anti-angiogenic effect [127,128,129]. Its mechanism of action can be elucidated from three aspects: animal models, cell signaling pathways, and network regulation.

At the animal model level, Jung et al. [12] found in a mouse model of OIR that kaempferol, as one of the active ingredients of *Aucuba japonica* extract, significantly reduces VEGF-induced retinal vascular leakage, confirming its anti-angiogenic efficacy at the overall level. At the cellular signaling pathway level, Xu et al. [85] revealed in HRECs under high-glucose conditions that kaempferol targets VEGF and placental growth factor (PGF), inhibits phosphoinositide 3-kinase (PI3K) expression and the phosphorylation of proto-oncogene tyrosine-protein kinase (Src), protein kinase B alpha (Akt1), and extracellular signal-regulated kinase (ERK1/2), thereby blocking the Src/Akt1/Erk1/2 signaling axis, suppressing cell proliferation, migration, and tube formation, and exerting anti-angiogenic effects, as shown in Figure 5C. At the network regulation level, Lin et al. [130] predicted through network pharmacology analysis that kaempferol is one of the core components of *Astragalus membranaceus* responsible for its anti-DR effect, involving multiple targets such as VEGFA, cyclooxygenase-2(COX-2), IL-6, and C-C motif chemokine ligand 2(CCL2). KEGG pathway enrichment analysis suggests that the advanced glycation end products–receptor for advanced glycation end products (AGE-RAGE) signaling pathway may play a key role. In vitro experiments further confirmed that kaempferol dose-dependently downregulates the expression of these target genes in human RPE cells (ARPE-19).

In summary, kaempferol exerts anti-angiogenic effects by modulating VEGF/PGF-associated signaling and suppressing the downstream Src/Akt1/ERK1/2 signaling axis, thereby inhibiting endothelial cell proliferation, migration, and tube formation. Network-based analyses further suggest that the AGE-RAGE pathway may contribute to the regulation of angiogenic and inflammatory mediators. However, the current in vivo anti-angiogenic evidence is restricted to the mouse OIR model, and validation in large-animal or chronic neovascularization models has yet to be undertaken. At present, the relative contributions of the various pathways still need to be verified using gene knockout or specific inhibitors. Future studies should explore combination treatment strategies with anti-VEGF drugs.

### 4.5. Immunomodulatory Effects on Microglia and Macrophage Polarization

The homeostasis of the immune system depends on the dynamic balance between the functions of immune cells. As a multifaceted immunomodulator, kaempferol can not only broadly regulate the activation and function of various immune cells [131,132,133] but also exert bidirectional regulation depending on the immune status of the body [134,135]. In the context of ocular disease treatment, kaempferol has been found to promote the anti-inflammatory phenotype of microglia on the one hand, while inhibiting the pro-inflammatory polarization of macrophages on the other. This section will elaborate on the regulatory mechanisms for these two cell types separately.

Regarding microglial regulation, Albalawi et al. [107] used a type 1 diabetic rat model together with in vitro microglial and macrophage experiments and found that kaempferol promotes the synthesis of arginase-1(Arg-1) and HO-1 in microglia, induces their polarization toward the M2 anti-inflammatory phenotype, achieves functional remodeling of microglia, and effectively protects retinal structure. Regarding macrophage regulation, Tian et al. [15] used a Wistar rat corneal allograft transplantation model and an LPS-induced human monocytic leukemia cell line (THP-1)-derived macrophage inflammation model. Through intraperitoneal injection of kaempferol and intervention with the autophagy inhibitor 3-methyladenine, combined with TUNEL staining, qPCR, and Western blot analysis, they confirmed that kaempferol induces autophagy (as shown in Figure 5D), thereby inhibiting NLRP3 inflammasome activation and M1 macrophage polarization, and consequently reducing corneal transplant rejection.This study thus reveals an interlocking cross-talk axis in which NLRP3-driven M1 polarization and IL-1β secretion bridge innate immune signaling to inflammatory cascades.

Taken together, in the treatment of eye diseases, kaempferol exerts immunoprotective effects by regulating microglia and macrophages. However, clinical translational research is currently lacking, and the current immunological evidence is restricted to rodent corneal transplantation and diabetic models, with no data from large-animal or chronic immune-mediated ocular disease models.”and its effects on other immune cells remain to be elucidated.

## 5. Safety Evaluation

The safety of kaempferol in ocular applications can be evaluated from four dimensions: local ocular toxicity, cellular toxicity, systemic toxicity, and genotoxicity.

In terms of local ocular toxicity, Chen et al. [18] prepared eye drops by combining kaempferol (1 μM) with ferulic acid (100 μM). After topical administration to rabbit eyes, no eye irritation was observed, and the corneal thickness of the treatment group was comparable to that of normal eyes. Meanwhile, at this concentration, no cytotoxicity was observed in human corneal epithelial cells cultured continuously for three days, and live/dead staining further confirmed its non-toxicity. In terms of cellular toxicity, Ondricek et al. [110] used RGC-5 retinal ganglion cells as a model and found that kaempferol reduced iodoacetic acid-induced caspase activation and ROS production, and rescued cell death, indicating that it does not produce additional cytotoxicity to neurons at protective concentrations. In terms of systemic toxicity, Yangzom et al. [136] conducted a 28-day oral administration study in mice to evaluate the effects of different doses of kaempferol on body weight, organ coefficients, hematological parameters, biochemical markers of liver and kidney function, and oxidative stress parameters. They also performed histopathological examinations of the heart, liver, kidneys, and lungs. The results showed no significant abnormalities in any of the indicators compared with the control group, and no morphological changes were observed. In terms of genotoxicity, Inthachat et al. [137] used the Ames test to detect extracts of kaempferol-rich ferns, and the result was negative, indicating no mutagenicity.

Overall, existing evidence suggests that kaempferol has a favorable safety profile at the local ocular, cellular, systemic, and genetic levels. However, direct research on the ocular safety of kaempferol is still relatively limited. The above evidence is derived mostly from in vitro cell experiments, short-term animal studies, or non-ocular administration routes, and there is a lack of long-term toxicity assessment and clinical human data.Moreover, the potential for cumulative toxicity or delayed adverse effects following chronic ocular use has not been evaluated in any preclinical model. Therefore, while current findings are encouraging, they should be interpreted with caution, and comprehensive long-term safety evaluations are indispensable prior to any consideration of clinical translation.

## 6. Conclusions and Future Perspectives

This review provides an integrated overview of the current understanding of kaempferol in ophthalmic applications, including its natural sources, structural characteristics, delivery strategies, disease-related effects, molecular mechanisms, and safety considerations. Accumulating evidence indicates that kaempferol is a multifunctional flavonol compound with anti-inflammatory, antioxidant, metabolic regulatory, anti-angiogenic, and immunomodulatory activities, which collectively contribute to its protective effects in experimental models of AMD, DR, diabetic cataract, dry eye disease, corneal neovascularization, fungal keratitis, corneal transplantation rejection, glaucoma, and retinoblastoma. Nevertheless, the current evidence mainly supports the biological activity of kaempferol rather than its therapeutic feasibility. The transition from experimental observations toward clinical development requires further clarification of disease relevance, delivery efficiency, mechanistic causality, pharmacokinetic behavior, safety profiles, and molecular optimization.

(i) Disease models

The current evidence base for kaempferol in ophthalmology has been largely established through in vitro studies and rodent models. While these models have provided important insights into potential protective mechanisms, their ability to predict therapeutic efficacy in human diseases remains limited. A major challenge is that many reported effects were obtained from acute injury or short-term intervention models, whereas major ophthalmic disorders such as AMD, DR, and glaucoma develop through prolonged and complex pathological processes. Consequently, the current literature provides strong support for biological activity but insufficient evidence regarding long-term efficacy, disease-stage responsiveness, and therapeutic durability. Future investigations should place greater emphasis on chronic and clinically relevant models, including large animal validation where appropriate, to determine the translational relevance of kaempferol and define parameters such as therapeutic windows and dose–response relationships.

(ii) Delivery systems

For ocular therapeutics, pharmacological activity alone does not guarantee therapeutic success, as achieving sufficient drug exposure at the target tissue remains a fundamental requirement. Current kaempferol delivery platforms, including gelatin nanoparticles, porous BSA membranes, platelet-derived extracellular vesicles, and polyvinylpyrrolidone-based nanocomposites, have demonstrated preliminary improvements in ocular retention and corneal penetration, particularly in anterior segment disease models. However, the current delivery landscape remains dominated by ocular surface applications, while strategies capable of achieving sustained and effective exposure in posterior segment tissues are still insufficiently explored. Future progress will depend on the development of delivery systems that overcome anatomical barriers, enhance retinal availability, and provide controlled release profiles. Surface-functionalized nanocarriers, microenvironment-responsive platforms, and clinically feasible administration routes represent potential directions for expanding kaempferol-based therapies toward retinal diseases.

(iii) Mechanistic networks

The expanding number of signaling pathways associated with kaempferol reflects its broad biological activity but does not necessarily indicate a complete mechanistic understanding. Current studies have linked kaempferol with multiple regulatory processes, including oxidative stress control, inflammatory signaling, angiogenesis inhibition, metabolic regulation, and immune modulation. The eight crosstalk relationships summarized in Figure 4 provide an integrated perspective on these interactions; however, most evidence has been generated from different cell types and experimental systems rather than through direct validation within a unified disease context. Therefore, these relationships should currently be interpreted as a conceptual framework rather than a fully established regulatory network. Future studies integrating multi-omics analysis, cell-specific approaches, and causal validation strategies will be essential for identifying key molecular drivers, cellular targets, and disease-specific mechanisms underlying kaempferol activity.

(iv) Clinical translation

Although current studies suggest a generally favorable safety profile of kaempferol at ocular, cellular, systemic, and genetic levels, the available evidence remains insufficient to support clinical application. The major translational gap lies in the limited understanding of the relationship among formulation, ocular exposure, efficacy, and long-term safety. In particular, ocular pharmacokinetic data remain scarce, prolonged toxicity evaluation is lacking, and potential interactions with established ophthalmic therapies, such as anti-VEGF agents and corticosteroids, have not been adequately characterized. Addressing these issues will require systematic pharmacokinetic studies using clinically relevant formulations, standardized long-term safety assessments, and preliminary evaluation of combination treatment strategies. Such evidence will be critical for determining whether kaempferol can advance beyond experimental validation toward therapeutic development.

(v) Chemical optimization

The therapeutic potential of kaempferol is closely associated with its diverse biological activities, but its intrinsic physicochemical limitations remain major obstacles for further development. Poor aqueous solubility, instability under alkaline conditions, and limited ocular bioavailability restrict its effective application despite encouraging pharmacological findings. Existing approaches, including glycosylation, methylation, sulfonation, prodrug strategies, and cocrystal formation, have provided potential solutions but remain at an early exploratory stage. Future development should not only focus on improving delivery of the parent compound but also consider rational optimization of kaempferol-derived molecules. Integration of structure–activity relationship studies, target identification, and computer-aided drug design may facilitate the generation of derivatives with improved pharmacological properties and translational potential.

In conclusion, kaempferol represents a promising bioactive molecule with broad therapeutic relevance across experimental ocular disease models. However, the current research landscape remains characterized by a gap between demonstrated pharmacological effects and clinically meaningful therapeutic evidence. The future development of kaempferol in ophthalmology will depend not on further accumulation of descriptive efficacy studies alone, but on establishing stronger links between disease models, drug exposure, molecular mechanisms, safety evaluation, and chemical optimization. Addressing these interconnected challenges will determine whether kaempferol can ultimately evolve from an experimental compound into a clinically valuable ophthalmic therapeutic.

## Figures and Tables

**Table 1:** Comparative Summary of Formulation Parameters and Ocular Performance of Kaempferol-Loaded Ocular Delivery Systems.

Parameter	GNP-KA	BSA Fibrous Film	PEV-KM	17PF-KAE
Carrier and formulation design	Type A gelatin NPs; two-step desolvation, 0.4% GA cross-linked; eye drops	BSA porous fibrous film; solution casting, KAE adsorbed (KAE: BSA = 0.05/0.13/0.25); therapeutic contact lens	Platelet-derived EVs; co-incubation loading; eye drops	PVP K-17PF; spontaneous aqueous complexation, optimized drug/polymer 18: 1 (*w*/*w*); eye drops
Size-related parameters	Particle size: 85–150 nm	Film thickness: ~17 μm	Particle size: 161.5 ± 29.5 nm	Particle size: 8.628 ± 0.066 nm
Zeta potential	+22 to +26 mV	Not applicable (film)	−13.7 ± 0.8 mV	Near neutral
Loading mechanism	Physical entrapment during desolvation/GA cross-linking	Adsorption onto film surface and internal porous network	Co-incubation loading; co-localization microscopy confirmed KM incorporation	Hydrogen bonding and hydrophobic interactions suggested by characterization
EE/Complexation efficiency	90–98%	Not applicable (adsorption); loading capacity: 86 mg/g (8.6%) at KAE: BSA = 0.25	61.0 ± 5.2%	Complexation efficiency: 64.3% (6: 1) → 92.8% (15: 1) → 93.1% (18: 1)
Loading capacity	4.6 ± 0.1% *w*/*w*	86 mg/g (8.6%)	Not explicitly reported as %	Not reported as %
Release behavior	Not studied	Sustained release >340 h (~14 d) in artificial tears; initial burst + sustained phase	PBS (pH 7) + 15% ethanol: 13.3% at 24 h (vs. free KM 75.8%)	Aqueous nanocomplex; release governed by dissociation equilibrium; no release curve reported
Storage stability	Not reported	Not explicitly reported	EV integrity (TSG101/ALIX/FLOT1) validated; long-term stability after loading not reported	90.7% at 4 °C, 87.1% at 25 °C after 6 wk
Corneal penetration	Not directly measured; in vivo efficacy in corneal NV model provides indirect evidence	Qualitatively enhanced vs. free KAE suspension; Papp not reported	Prolonged retention infers enhanced penetration; Papp not reported	Papp: 4.07 × 10^−7^ vs. free KAE 2.10 × 10^−7^ cm/s (*p* < 0.05)
Ocular retention	TAMRA-labeled GNPs detectable on ocular surface by IVIS; quantitative duration not reported	Film remained on corneal surface, replaced every 24 h	~80% at 10 min → ~50% at 50 min vs. free KM 30% → 11.2% (*p* < 0.0001)	PVP mucoadhesion may contribute; quantitative retention data not reported
Biocompatibility	Gelatin GRAS; 250 μg/mL showed short-term cytotoxicity	Hemolysis < 1%; no cytotoxicity in HCEC	Platelet-derived origin with potential biocompatibility advantages	Cell viability > 94%; Draize test: no ocular irritation
Advantages	Highest EE (90–98%); biodegradable gelatin Positively charged surface may improve ocular retention	Ultra-long sustained release (>14 d); high transparency (~96%) Supports corneal epithelial healing; excellent hemocompatibility	Natural vesicle platform with platelet-derived surface markers Best-documented ocular retention; sustained release	Ultra-small size (~9 nm); good aqueous dispersibility Only system with quantified Papp and storage stability; no irritation
Limitations	GA cross-linking: potential residual toxicity concerns Release kinetics, Papp, and storage stability not characterized	Film format less conventional than eye drops Initial burst release; Papp and storage stability not reported	Lower EE (~61%); preparation complexity Loading optimization and Papp/stability data limited	Simple complexation rather than encapsulation Release curve and in vivo retention/pharmacokinetic data unavailable
In vivo efficacy	Mouse corneal alkali-burn NV model; eye drops (5 μL/eye, q.d., 7 d); reduced vessel growth, MMP and VEGF	Mouse corneal alkali-burn model; film replaced q24h; promoted epithelial healing, reduced IL-1β, inhibited NV	Mouse corneal alkali-burn CoNV model; 1% PEV eye drops; reduced angiogenesis and related gene expression	Rabbit absorption + mouse alkali-burn model; increased KAE in cornea/aqueous humor; improved healing vs. free KAE and HA
Ref.	[16]	[31]	[32]	[57]

Note: GNP-KA, gelatin nanoparticles loaded with kaempferol; BSA, bovine serum albumin; PEV-KM, platelet-derived extracellular vesicles loaded with kaempferol; 17PF-KAE, polyvinylpyrrolidone K-17PF and kaempferol nanocomplexes; GA, glutaraldehyde; NPs, nanoparticles; EVs, extracellular vesicles; PVP, polyvinylpyrrolidone; EE, encapsulation efficiency; Papp, apparent permeability coefficient; GRAS, generally recognized as safe; HCEC, human corneal epithelial cells; *TSG101*, tumor susceptibility gene 101; *ALIX*, ALG-2-interacting protein X; *FLOT1*, flotillin-1; IVIS, in vivo imaging system; TAMRA, carboxytetramethylrhodamine; NV, neovascularization; CoNV, corneal neovascularization; VEGF, vascular endothelial growth factor; MMP, matrix metalloproteinase; IL-1β, interleukin-1 beta; HA, sodium hyaluronate; KM, kaempferol.

## Data Availability

No new data were created or analyzed in this study. Data sharing is not applicable to this article.

## Abbreviations

The following abbreviations are used in this manuscript:

17PF-KAE	ultra-small nanocomposites based on polyvinylpyrrolidone K-17PF and kaempferol
ADAMTS-1	a disintegrin and metalloproteinase with thrombospondin motifs 1
AGEs	advanced glycation end products
Akt	protein kinase B alpha
AR	aldose reductase
Arg-1	arginase-1
BSA	bovine serum albumin
CCK-8	Cell Counting Kit-8
CCL2	C-C motif chemokine ligand 2
CD54	intercellular adhesion molecule-1
COX-2	cyclooxygenase-2
DPPH	2,2-diphenyl-1-picrylhydrazyl
DR	diabetic retinopathy
ERK	extracellular signal-regulated kinase
ERRα	estrogen-related receptor alpha
EVs	extracellular vesicles
GNP-KA	kaempferol-loaded gelatin nanoparticles
GLP	Good Laboratory Practice
HO-1	heme oxygenase-1
HRECs	human retinal endothelial cells
IL-1β	interleukin-1 beta
IL-6	interleukin-6
IRF7	interferon regulatory factor 7
JNK	c-Jun N-terminal kinase
LDH	lactate dehydrogenase
LPS	lipopolysaccharide
MAPK	mitogen-activated protein kinase
NF-κB	nuclear factor kappa-B
NLRP1	NLR family pyrin domain containing 1
NLRP3	NLR family pyrin domain containing 3
NMR	Nuclear Magnetic Resonance
Nrf2	nuclear factor erythroid 2-related factor 2
OIR	oxygen-induced retinopathy
PEV-KM	platelet-derived extracellular vesicles loaded with kaempferol
PGF	placental growth factor
PI3K	phosphoinositide 3-kinase
pRb	retinoblastoma protein
RAGE	receptor for advanced glycation end products
RLAR	rat lens aldose reductase
ROS	reactive oxygen species
RPE	retinal pigment epithelial
SAR	structure-activity relationship
Src	proto-oncogene tyrosine-protein kinase
ssDNA	single-stranded DNA
STAT1	signal transducer and activator of transcription 1
TGF-β1	transforming growth factor-beta 1
TNF-α	tumor necrosis factor-alpha
TSP-1	thrombospondin-1
TUNEL	terminal deoxynucleotidyl transferase dUTP nick end labeling
VEGF	vascular endothelial growth factor
